# ANDSystem: an Associative Network Discovery System for automated literature mining in the field of biology

**DOI:** 10.1186/1752-0509-9-S2-S2

**Published:** 2015-04-15

**Authors:** Vladimir A Ivanisenko, Olga V Saik, Nikita V Ivanisenko, Evgeny S Tiys, Timofey V Ivanisenko, Pavel S Demenkov, Nikolay A Kolchanov

**Affiliations:** 1 The Institute of Cytology and Genetics, The Siberian Branch of the Russian Academy of Sciences, Novosibirsk, Russia; 2 PB-soft, Llc, Novosibirsk, Russia; 3 Novosibirsk State University, Novosibirsk, Russia

**Keywords:** ANDSystem, ANDCell, ANDVisio, text mining, information extraction, natural language processing (NLP), associative networks, gene networks, disease and disorders

## Abstract

**Background:**

Sufficient knowledge of molecular and genetic interactions, which comprise the entire basis of the functioning of living systems, is one of the necessary requirements for successfully answering almost any research question in the field of biology and medicine. To date, more than 24 million scientific papers can be found in PubMed, with many of them containing descriptions of a wide range of biological processes. The analysis of such tremendous amounts of data requires the use of automated text-mining approaches. Although a handful of tools have recently been developed to meet this need, none of them provide error-free extraction of highly detailed information.

**Results:**

The ANDSystem package was developed for the reconstruction and analysis of molecular genetic networks based on an automated text-mining technique. It provides a detailed description of the various types of interactions between genes, proteins, microRNA's, metabolites, cellular components, pathways and diseases, taking into account the specificity of cell lines and organisms. Although the accuracy of ANDSystem is comparable to other well known text-mining tools, such as Pathway Studio and STRING, it outperforms them in having the ability to identify an increased number of interaction types.

**Conclusion:**

The use of ANDSystem, in combination with Pathway Studio and STRING, can improve the quality of the automated reconstruction of molecular and genetic networks. ANDSystem should provide a useful tool for researchers working in a number of different fields, including biology, biotechnology, pharmacology and medicine.

## Background

There is no doubt that one of the most important sources of reliable biological data is the scientific literature. The well-known PubMed database contains more than 24 million abstracts, which makes it extremely difficult for researchers to manually analyze such huge amounts of data. Text- and data-mining approaches can be used for the automated extraction of information from scientific literature. However, another problem is obtaining information in a compact and convenient format that is suitable for further analysis. One of the approaches to this challenge is to present the extracted data in the form of associative molecular genetic networks that describe various interactions between genes, proteins, metabolites, biological processes and diseases.

Pathway Studio [1], STRING [2], Biblio-MetReS [3], Meshop [4] and Coremine [5] are well-known examples of text-mining systems dedicated to the reconstruction of molecular-genetic networks. It should be noted that most of the programs based on automated text-analysis approaches mainly focus on findings of the interactions between the molecular and genetic objects themselves, without further classification of the interaction type, or the limitation of the classification to only a few basic types. At the same time, a detailed description of the molecular mechanisms of biological processes, which requires the consideration of a wide variety of relationships between molecular and genetic objects, is a necessary prerequisite for the majority of research studies. One of the possible solutions to this problem is the combined use of several programs that provide information about different types of molecular and genetic interactions, which can result in the reduction of the error rate related to the false extraction of information from text that can occur if each program is used separately. In this regard, the development of automated tools based on original text-mining methods allowing retrieval of an extended description of interactions compared with existing programs is a current topic of interest in the data-mining field.

Here, we describe for the first time the ANDSystem package, which is dedicated to the reconstruction of associative networks based on an automated analysis of scientific publications, while providing a wide range of types of interactions between molecular and genetic objects, diseases and pathways. Recently, ANDSystem was used for the reconstruction of the associative molecular genetic networks associated with various human diseases, including myopia and glaucoma [6], dilated cardiomyopathy [7], and bronchial asthma and tuberculosis [8]. In the case of asthma and tuberculosis, it was shown that the structure of molecular genetic networks describing molecular interactions between inversely comorbid diseases is significantly different from the same networks constructed for random pairs of diseases [8]. With the use of ANDSystem, a network analysis of proteomic data was performed. For example, molecular genetic networks were reconstructed describing the interactions between proteins identified in the urine of healthy humans in a 520-day isolation experiment [9] and for proteins differentially expressed in various *Helicobacter pylori *strains isolated from patients with chronic gastritis and gastric tumors [10].

## Implementation

### ANDSystem main modules

**Figure 1:**
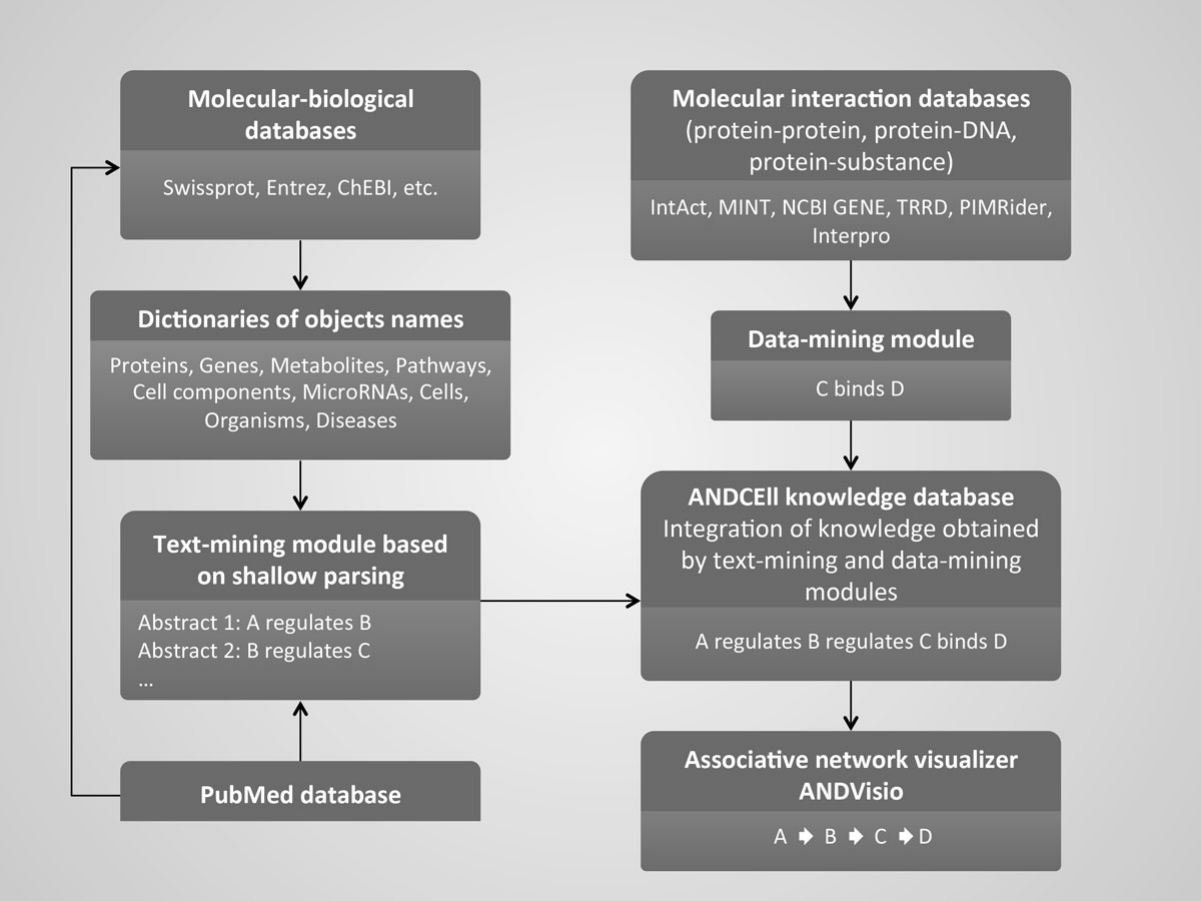
**Schematic illustrating literature and database mining implemented in ANDSystem**.

ANDSystem contains both server and client modules, including a knowledge extraction module, an ANDCell knowledge base and ANDVisio (Figure 1). The knowledge extraction module is used for formation and updating of the ANDCell knowledge base. ANDVisio is dedicated to the automated reconstruction and visualization of associative molecular genetic networks and is a client module of ANDSystem, while ANDCell and the knowledge extraction module are located on the server.

### Knowledge extraction module

This module is based on shallow parsing technology [11,12]. Its main elements are comprised of dictionaries and semantic templates. In ANDSystem, the following types of objects are presented: genes, proteins, microRNAs, metabolites, diseases, biological processes, cell components, cell lines and organisms. The formation of dictionaries for these object types was carried out in two stages. During the first stage, the extraction and normalization of names and synonyms of objects from external factual databases were performed. The following databases were used: SwissProt (dictionary of proteins); Entrez GENE (dictionary of genes); ChEBI (dictionary of metabolites); MESH (dictionary of diseases); MirBase (dictionary of microRNAs); Gene Ontology (dictionaries of pathways, cellular components and molecular functions; Cell Lines database (CLDB) (dictionary of cell names); and Entrez Taxonomy (dictionary of organisms).

Normalization is one of the most widely used approaches for the extension of dictionaries with synonyms [13]. Thus, in the second stage, a further expansion of the list of synonyms was performed by comparing the normalized forms of terms with the texts of scientific papers. The full algorithm included following steps:

1. Splitting of the full text into separate sentences.

2. Fragmentation of each sentence by utilizing a sliding window with variable length.

3. Normalization of text defined by the sliding window.

4. Comparison of normalized text with normalized names of objects. The name of an object is considered to be found if the normalized text is identical to the normalized name of the object.

5. Comparison of the non-normalized texts corresponding to the sliding window and the object name. If the initial texts are different from each other, the original text of the fragment is considered to be a new recognized synonym for the given name.

Name normalization was performed with the use of the following algorithm:

1. Conversion of text to one register.

2. Removal of punctuation and dashes.

3. Removal of articles such as "a," and "the," etc.

4. English transliteration of Greek letters.

5. Lemmatization based on context-free morphological analysis.

6. Sort a list of words alphabetically.

Thus, each normalized name was represented by the alphabetically ordered list of words in the normal form. As an example, consider the name of the biological process, "classic complement activation pathway" obtained from Gene Ontology (GO). In one of the papers [14], the authors made a transposition of words and added some prepositions and articles to generate the description "activation of the classic pathway of complement." However, after performing the normalization algorithm from above to the GO and authors' forms, they appeared to be completely identical to "activation classic complement pathway." Thus, our method had successfully identified the name of the process given in the paper as a synonym for the name obtained from the database.

A statistical summary of the dictionaries used in ANDSystem is given in Table 1. The largest dictionary is "Genes," while the smallest amount of objects was found in the "Cell components" dictionary. An expansion of the number of synonyms using our normalization algorithm increased the volume of dictionaries by an average of 31%.

**Table 1:** 

Dictionary topic	Number of unique names	Average number of synonyms per unique name
Proteins	233,158	7.7
Genes	1,938,128	2.1
Diseases	4,076	11.2
Metabolites	30,269	3.3
Pathways	57,877	2.3
Cell components	2,091	2.6
Cells	396,841	1
microRNA	4,517	1
Organisms	11,964	2.9

### Semantic templates

**Figure 2:**
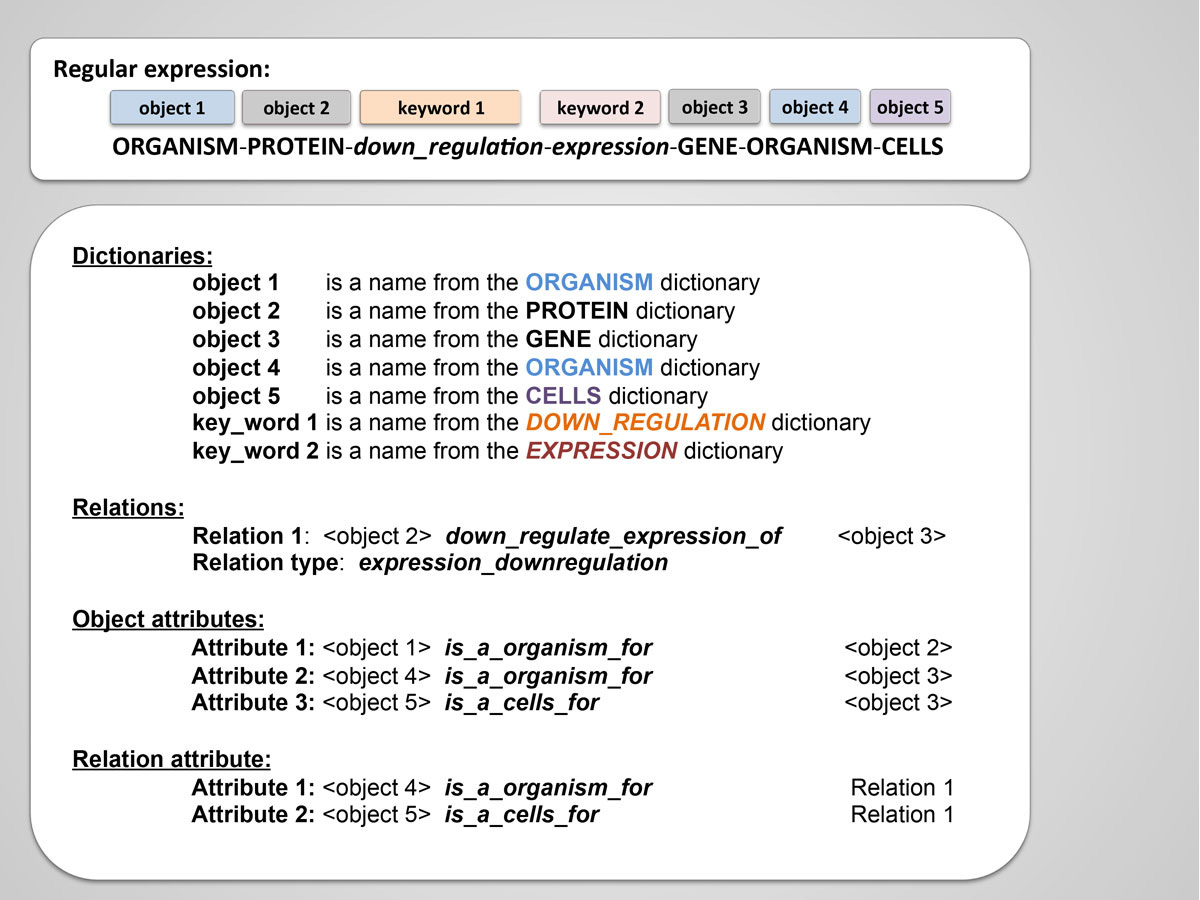
**An example of the ANDSystem semantic template**.

A semantic pattern is a structured record containing information about object types, dictionaries, text analysis rules or regular expressions and descriptions of the interaction semantics (see Figure 2). We have developed about 3000 semantic templates that allowed us to conduct automated knowledge extraction from texts of scientific publications with about 24 different types of interactions. These interaction types were suggested according to a manually conducted expert analysis of more than 5,000 PubMed abstracts. Each abstract contained the names of at least two molecular-genetic objects. Twenty three types of interactions were selected, allowing us to describe most of the interactions that were identified in the texts by experts. Additionally, we have added an "association" interaction type, describing the 23 types of selected interactions, as well as all those interactions that were not included in this number at the same time.

The structure of the template includes the following main fields: Regular Expression, Dictionaries, Relations, Object Attributes and Relation Attributes. A regular expression defines the order of the names of objects and special keywords, indicating the specified type of interaction between the objects in the analyzed sentence. The structure of the regular expression is a sequence of identifiers of dictionaries (dictionaries of both objects and keywords). The "-" symbol is used as a separator between these identifiers. The regular expression can also contain the information about allowable number of any words that can be placed between the names of objects in a sentence. Also, a regular expression may comprise a negation (i.e., any words of a sentence except those specified in curly brackets {} are allowed). For example, {metabolite} means that any object except those listed in the "Metabolite" dictionary is allowed. Figure 2 shows an example of the one of the ANDSystem templates. It contains object (organisms, proteins, genes, cells) and keyword (down-regulation, expression) dictionaries. According to the template, regular expression, it follows that object 2, which is any protein from the Proteins dictionary, negatively regulates the expression of object 3, which can be any gene from the Genes dictionary. Both the objects and interactions between these objects can have their own attributes. Object 1 is an organism for object 2 and object 4 for object 3. Thus, object 3 (gene) has an additional attribute - cell line (object 5), wherein this gene is expressed. Objects 4 and 5 are also attributes of the interaction, indicating an organism and cell line where this interaction takes place.

**Figure 3:**
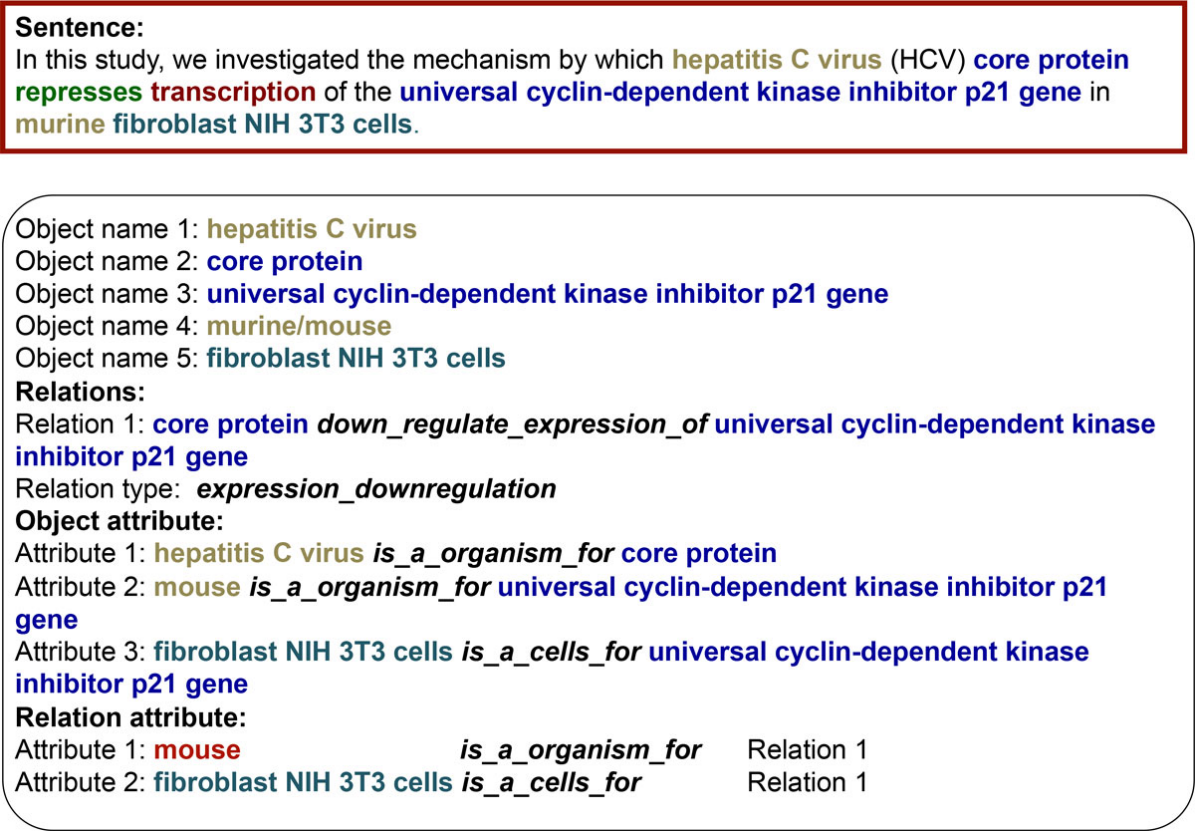
**An example of information retrieval using the ANDSystem template**.

The template contains information about the types of objects and types of their interactions without the specification of the object names. The match of the regular expression with the text of the sentence allows the identification of particular object names. In the case of the considered template (Figure 2), the object names were established from the following sentence (PubMed Id: 12185267): "*In this study, we investigated the mechanism by which hepatitis C virus (HCV) core protein represses transcription of the universal cyclin-dependent kinase inhibitor p21 gene in murine fibroblast NIH 3T3 cells*." (see Figure 3).

All of our templates are divided into several groups according to the type of interactions. In each group, the priorities are assigned to templates according to the hierarchical classification of templates based on their complexity. The simple templates that are assigned to the extraction of information about basic events have lower priority, while more complex templates that are designed to extract more specific information in addition to the basic data have a higher priority. For the template from above, there is a hierarchical group of patterns differing by both completeness of the retrieved information and priorities. The following regular expressions from this group of templates can be considered as examples:

1) **PROTEIN-*down_regulation-expression*-GENE;**

2) **PROTEIN-*down_regulation-expression*-GENE-ORGANISM;**

3) **ORGANISM-PROTEIN-*down_regulation-expression*-GENE-ORGANISM-CELLS.**

With the help of template 1, only the basic information about the regulation of gene expression can be extracted: *core protein represses transcription of the universal cyclin-dependent kinase inhibitor p21 gene*. Template 2 also provides information about the organism where the event was observed (*mouse *for this example). The most detailed data will be provided by the third template, which includes basic information, as well as information on the involved organisms (*hepatitis C virus *and *mouse) *and cell line *(fibroblast NIH 3T3)*. Only the third template will be considered by ANDSystem due to the highest priority. This approach can also be helpful in cases where participants of the interaction are mutants rather than native forms of proteins. For example, the sentence, "*Overexpression of dominant-negative forms of Ras or RhoA completely blocked PDGF-induced p27 (KIP1) degradation, but only dominant-negative Ras inhibited cyclin D1 protein expression*" (PubMed Id: 9407076) contains information that only the dominant-negative form of the Ras protein inhibits expression of the cyclin D1 protein. This event is described in the following sentence fragment: "*dominant-negative Ras inhibited cyclin D1 protein expression*." In particular, such a proposal can be performed by two types of templates:

1) **GENE-*inhibited*-GENE-*expression*;**

2) ***mutant*-GENE-*inhibited*-GENE-*expression***.

Template 1 will extract false information that Ras protein inhibits expression of the cyclin D1 gene because this template does not include additional information about the mutation. At the same time, pattern 2 will provide a correct statement that mutant Ras protein inhibits expression of cyclin D1. In this example, template 2 has a higher priority.

### The ANDCell knowledge base

In the current version of ANDSystem, about 15 million PubMed abstracts published in the period ranging from 1990 to 2013 were analyzed. The extracted information describing 5,395,313 interaction events and involving 452,209 objects was stored in ANDCell.

In addition to the data extracted from the texts of PubMed, ANDCell also contains information about the 905,799 interaction facts extracted from external databases, including protein-protein interactions from IntAct [15] and MINT [16], regulation of gene expression from TRRD [17], protein-pathway interactions from InterPro [18], protein expression from EntrezGene [19], microRNA-protein interactions from mirBASE [20], and involvement of proteins into pathways from UniProt-GOA [21]. A summary of ANDCell statistics are shown in Table 2.

**Table 2:** General statistics on ANDCell database content and descriptions of molecular-genetics interactions.

Interaction type	Involved objects	Description	Number of ANDCell entries
association	Proteins, genes, metabolites, cell components, diseases, pathways	Association type is used to define the relationships between genes and diseases. The Association is also used as a type of relationship between other objects, if a particular type of relationship has been omitted in the text.	3,433,168
involvement	Proteins, pathways	Involvement of proteins into pathways (UniProt-GOA).	728,947
interaction	Proteins, genes, metabolites, cell components	Formation of molecular complexes.	242,757
expression	Proteins, genes	The protein product of gene expression (NCBI gene)	178,761
expression regulation*	Proteins, genes	Direct regulation by a transcription factor that physically interacts with a gene promoter and indirect regulation of gene expression by proteins.	236,298
pathway regulation*	Proteins, metabolites, pathways	Activation and termination of pathway functioning.	234,179
transport regulation	Proteins, metabolites	Regulation of transport proteins or metabolites between cell compartments, as well as the secretion of these molecules from the cell.	64,810
treatment	Proteins, metabolites, diseases	The use of a molecular agent for treatment of a known disease.	51,195
catalyze	Proteins, metabolites	Catalytic reactions are reactions involving metabolites as substrates and products; also, a protein as an enzyme catalyzing this reaction.	49,173
activity regulation*	Proteins, metabolites, cellular components	Regulation of activity/function of proteins and cellular components.	101,953
degradation regulation*	Proteins, metabolites, cellular components	Regulation of stability or degradation of molecular objects.	17,751
miRNA regulation	miRNA, proteins	Regulation of protein expression.	23,576
coexpression	Genes	Co-expression of several genes.	6,618
cleavage	Proteins	Protein cleavage events. Protein substrate and proteolytic enzyme are participants.	2,178
catalyzed modification	Proteins, metabolites	Catalysis of post-translational protein modifications.	430
conversion	metabolites	Catalytic reaction in a case when a catalyst enzyme is not indicated; also when the reaction proceeds without a catalyst.	23,519

* three types of regulations (regulation, upregulation and downregulation) are presented for this group.

In addition to information about the interactions, ANDCell contains data about organisms and cells in which the interactions were observed.

**Figure 4:**
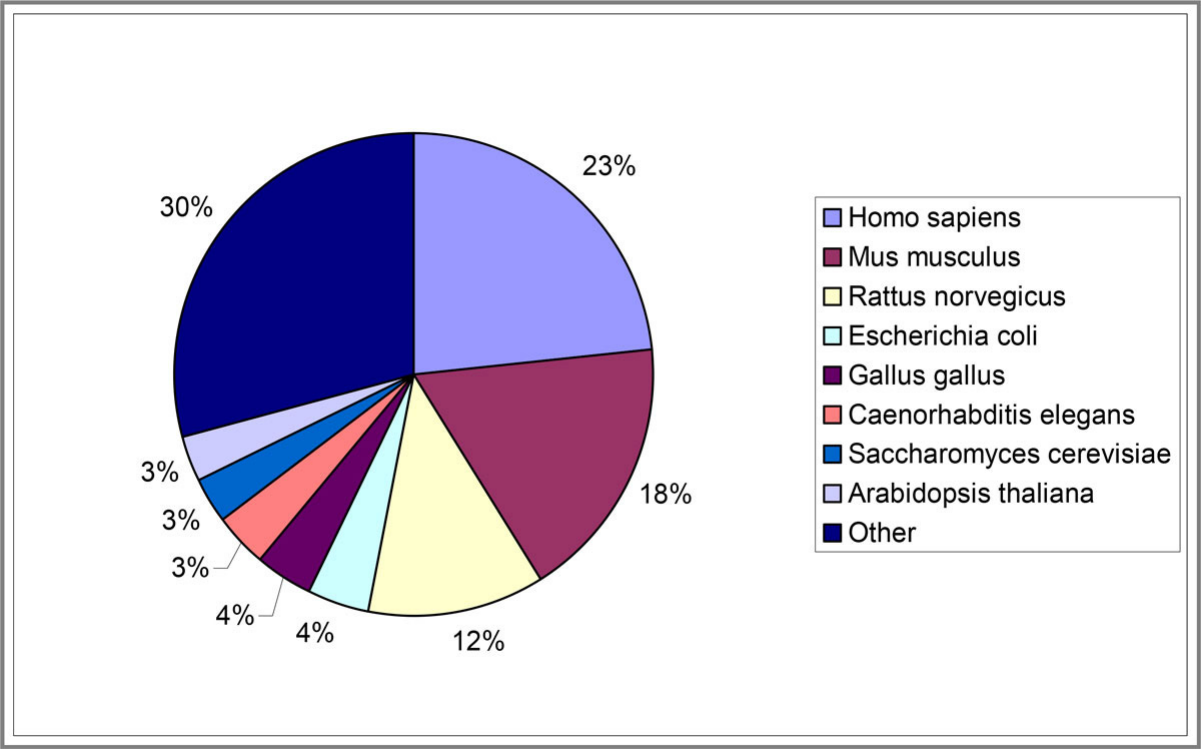
**Distribution of the number of interactions for the 8 most represented in ANDCell organisms**.

In ANDCell, 17,413 organisms are described, with *Homo sapiens, Mus musculus *and *Rattus norvegicus *being the most highly represented organisms (Figure 4).

### The ANDVisio program

ANDVisio is a client module of ANDSystem which allows one to perform user queries to the ANDCell knowledge base and provides reconstruction, analysis and visualization of molecular genetic networks (associative networks) in the form of bipartite graphs based on these queries. Vertices of such graphs represent the objects and edges represent the interactions between them. For the reconstruction of associative networks the user can set one or more names of objects of interest (genes, proteins, metabolites, organism, etc.). Also, additional sets of parameters can be specified, including information sources about interactions, interaction types and object types.

**Figure 5:**
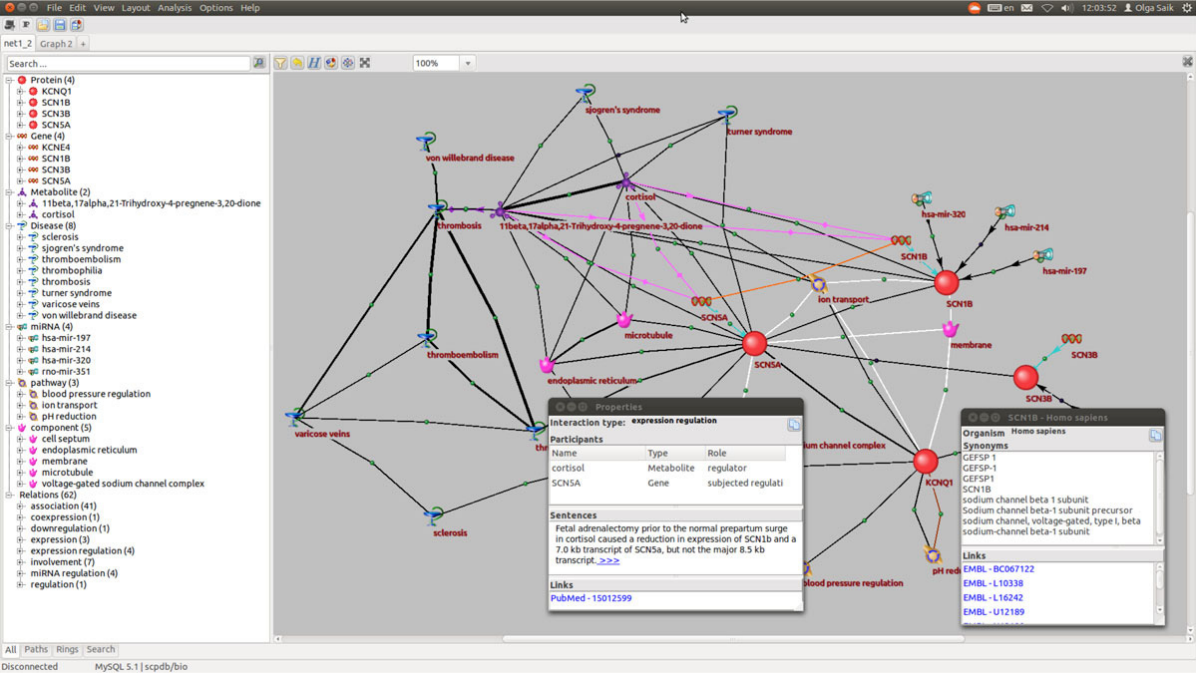
**The ANDVisio interface**.

The ANDVisio interface containing a fragment of the network associated with cardiovascular human diseases that describes the interaction between diseases, pathways, microRNAs, proteins, genes and metabolites is shown in Figure 5. ANDVisio provides editing, search and saving of associative networks in different formats. Also, ANDVisio is equipped with various tools supporting a number of different functions, such as filtering by object types, relationships between objects and information sources, as well as the generation of various graph layouts, including the capability to search the shortest pathways and cycles. A detailed description of ANDVisio can be found in reference [22].

## Results and Discussion

**Figure 6:**
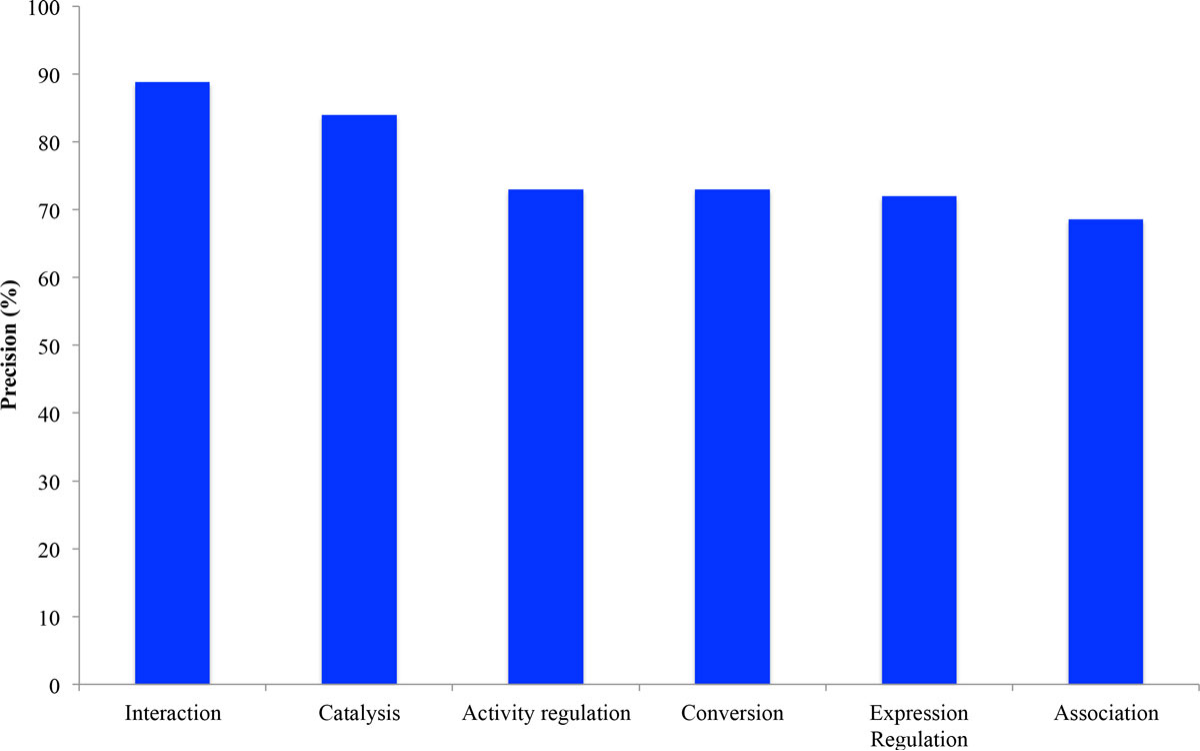
**Precision values for the six main types of ANDSystem interactions**.

To estimate the quality of data involving the interactions identified in the ANDCell knowledge base of ANDSystem, precision and recall values were calculated. Precision was estimated as the ratio of the number of correctly identified interactions to the total number of interactions in the testing set. The testing set was prepared as a number of interactions randomly selected from ANDCell. We estimated precision values for the 6 main types of ANDSystem interactions, including "interaction," "catalysis," "activity regulation," "conversion," "expression regulation" and "association," covering about 90% of all molecular-genetic interactions described in ANDCell (Figure 6). We did not consider "involvement" and "expression" in the accuracy estimation, because all the data describing these interaction types was extracted from the UniProt-GOA and NCBI Gene databases, respectively. For each type of interaction a testing set consisted of 100 unique interactions.

True and false interactions were classified manually by experts. The error was defined as a wrongly recognized name of at least one of the participants of the interaction or as an incorrectly established interaction between them. The maximum and minimum precision for "interaction" and "association" types were found to be 88.8% and 68.6%, respectively. The average precision was calculated to be 76.5%. It should be noted that the "association" is used in ANDSystem to determine the relationship between a pair of objects in a case when a more specified type of interaction was not identified. In this regard, a low precision value for the "association" is caused by failing to use strict templates for this type of interaction.

For the assessment of recall values, a Gold Standard containing expertly verified information about different types of molecular-genetic interactions extracted from the GeneNet database [23-25] was created. We used a GeneNet database as a source of information for our Gold Standard due to the fact that it was manually created by experts on the basis of scientific publications without the use of any automated text-mining tools. The Gold Standard was formed on the basis of 17 randomly taken GeneNet networks containing a total of 2,286 interactions between genes, proteins and metabolites. To establish one-to-one correspondence between GeneNet and ANDSystem, only interactions with the following identifiers were considered: SWISS-Prot for proteins, ENTREZ_GENE for genes and the CAS number for metabolites. Using these criteria, 741 interactions remained in the Gold Standard, including 730 participants (349 proteins, 23 genes and 358 metabolites). ANDCell contained 398 interactions from this Gold Standard. Thus, the recall value for ANDSystem was about 54%. In order to compare ANDSystem with existing programs, we applied our Gold Standard to well-known text-mining based systems, such as Pathway Studio [1] and STRING [2]. Surprisingly, the recall for Pathway Studio did not exceed 22%. It was found that some proteins from the SWISS-Prot database were not identified in the Pathway Studio. Out of 349 proteins involved in 741 interactions of our Gold Standard, only 96 proteins involved in 167 interactions were identified in Pathway Studio. The recall value for Pathway Studio calculated from these 167 interactions was 94%. It was also found that recall for ANDSystem calculated with the same sample appeared to be 84% (146 interactions were found out of 167), which is slightly inferior to this well-known program. To apply our Gold Standard to STRING, we left interactions involving proteins only and identified 31 out of 97 interactions (32% recall). It should be mentioned that the threshold of significance in STRING (the parameter for searching interactions) was set as "high," because unlike Pathway Studio and ANDSystem, this program is based on the co-occurrence approach.

**Figure 7:**
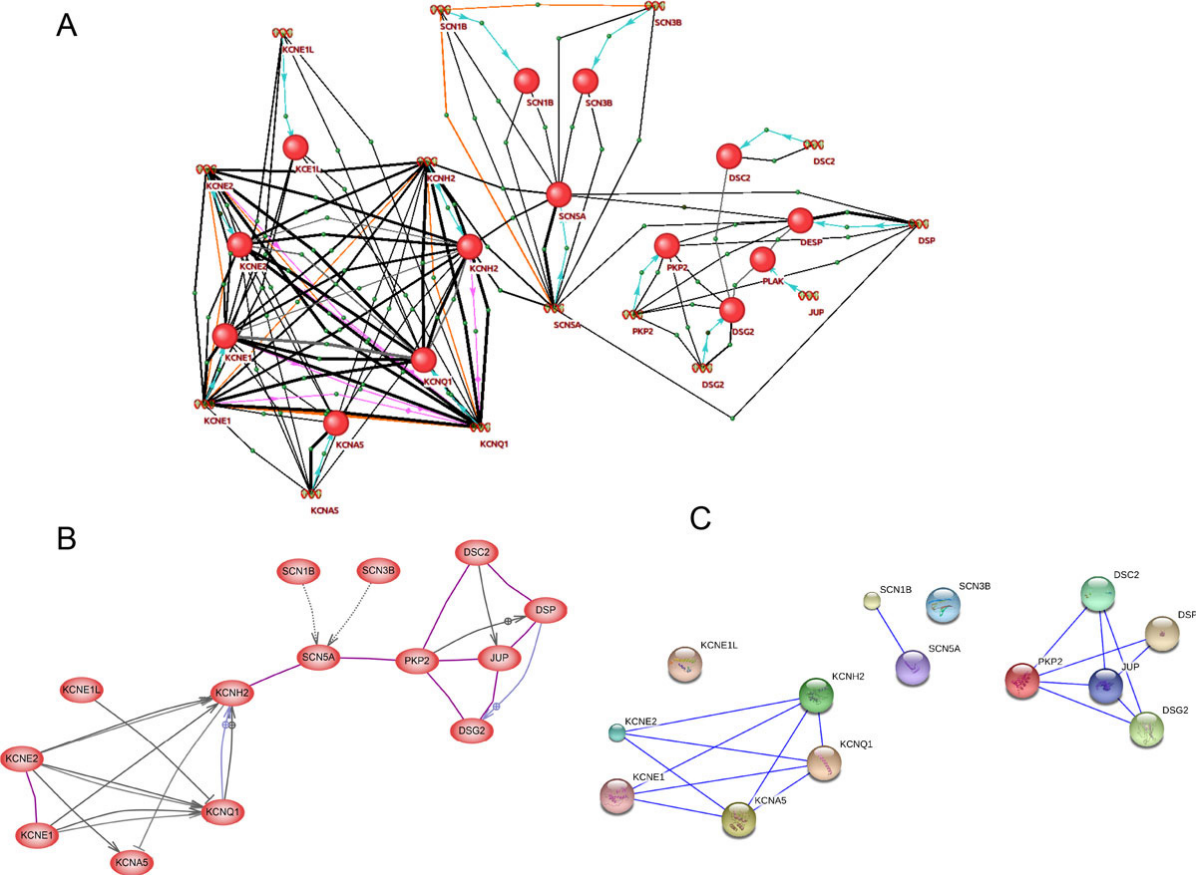
**An example of interaction networks reconstructed with ANDSystem (A), Pathway Studio (B) and STRING (C)**. The networks were reconstructed for 14 genes/proteins participating in the ≪regulation of heart rate by cardiac conduction≫ of the Gene Ontology biological process (GO: 0086091). Proteins are presented with red balls and genes with double helixes in the ANDSystem network. For Pathway Studio and STRING networks, proteins are presented with red ovals and colored balls, respectively.

It can be expected that the combined use of programs based on different text-mining methods can increase the completeness of the description of the molecular interactions in the studied biological processes. We compared the completeness of the ANDSystem, Pathway Studio and STRING networks by applying these programs to the automated reconstruction of networks describing interactions between 14 randomly selected genes from the Gene Ontology biological process, 蠐regulation of heart rate by cardiac conduction≫ (GO: 0086091), which plays an important role in the functioning of the cardiovascular system (see Figure 7). The ANDSystem network includes 112 interactions for 39 pairs of objects. The network contains the following interaction types: 14 蠐expression≫, 5 ≪expression regulation≫, 7 ≪coexpression≫, 8 ≪interaction≫, and 78 ≪association≫ (Figure 7A). The Pathway Studio network contains 26 interactions for 22 pairs of objects, including the following interaction types: 9 ≪Binding≫, 9 ≪DirectRegulation≫, 2 ≪Expression≫, 4 ≪MolTransport≫ and 2 ≪Regulation≫ (Figure 7B). The STRING network contains 18 ≪Binding≫ interactions for 18 pairs of objects (confidence (score) = 0.900) (Figure 7C). In the ANDSystem network, genes and proteins are presented as separate objects, while in STRING and Pathway Studio networks these types of objects are united. To compare ANDSystem with Pathway Studio and STRING, we converted the ANDSystem network into a network in which genes and proteins were also presented as one object. After such a procedure, the number of interactions in the ANDSystem network appeared to be 88 interactions for 21 pairs of objects. Fourteen ≪expression≫ interactions between genes and proteins (products of their expression) were deleted. Also, 10 ≪association≫ interactions were removed because both participants (genes and proteins) had the same interactions.

Thus, the combined ANDSystem/Pathway Studio/STRING network contains 28 pairs of interacting objects (with 12 pairs among them being the same for each system), while, the combined Pathway Studio/ANDSystem, STRING/ANDSystem and Pathway Studio/STRING networks contain 16, 14 and 15 shared pairs of interacting objects, respectively. The 3 pairs of interacting objects were found only in the ANDSystem network, including the following interaction types: 6 ≪association≫ and 1 ≪coexpression≫. The Pathway Studio network contained 3 unique pairs of interacting objects including 2 ≪Binding≫ and 1 ≪Expression≫. The STRING network contained only 1 unique pair of interacting objects. Thus, the combined use of different text-mining based systems can help to obtain the most detailed information about molecular genetic interactions.

## Conclusion

ANDSystem, which has the automated capability of reconstructing networks, was developed for the purpose of scanning literature to extract relationships between diseases, pathways, cell components, proteins, genes, microRNAs and metabolites. ANDSystem incorporates utilities for automated knowledge extraction from PubMed and analysis of factographic databases. ANDSystem accuracy is comparable to the accuracy of well-known text-mining systems, such as Pathway Studio and STRING, but in contrast to these systems, it provides a more detailed description of interactions between molecular and genetic objects. It is obvious that existing text-mining systems cannot extract full information about molecular-genetic interactions contained in scientific publications. In this study, we demonstrated that the combined use of ANDSystem with Pathway Studio and STRING reduced the information loss that results from using each system individually.

## Availability and requirements

ANDSystem is freely available under the following link: http://pbiosoft.com/andsystem

## Competing interests

The authors declare that they have no competing interests.

## Authors' contributions

VAI, NVI and TVI developed text mining methods, algorithms and software for ANDSystem, PSD developed the ANDVisio program and a structure for the ANDCell database, OVS and EST provided evaluation of the accuracy of ANDSystem and its comparison with existing programs. NAK was the overall director of the research, and contributed to the writing and editing of this manuscript. All authors read and approved the final manuscript.

## Declarations

Publication of this article has been funded by Russian Science Foundation grant No 14-24-00123

This article has been published as part of *BMC Systems Biology *Volume 9 Supplement 2, 2015: Selected articles from the IX International Conference on the Bioinformatics of Genome Regulation and Structure\Systems Biology (BGRS\SB-2014): Systems Biology. The full contents of the supplement are available online at http://www.biomedcentral.com/bmcsystbiol/supplements/9/S2.
